# Integrative approach for inference of gene regulatory networks using lasso-based random featuring and application to psychiatric disorders

**DOI:** 10.1186/s12920-016-0202-9

**Published:** 2016-08-10

**Authors:** Dongchul Kim, Mingon Kang, Ashis Biswas, Chunyu Liu, Jean Gao

**Affiliations:** 1 Department of Computer Science, University of Texas Rio Grande Valley, Edinburg, 78541 TX US; 2 Department of Computer Science, Kennesaw State University, Marietta, 30144 GA US; 3 Department of Computer Science and Engineering, University of Texas at Arlington, Arlington, 76019 TX US; 4 Department of Psychiatry, University of Illinois at Chicago, Chicago, 60607 IL US

**Keywords:** Gene regulatory network, Psychiatric disorder

## Abstract

**Background:**

Inferring gene regulatory networks is one of the most interesting research areas in the systems biology. Many inference methods have been developed by using a variety of computational models and approaches. However, there are two issues to solve. First, depending on the structural or computational model of inference method, the results tend to be inconsistent due to innately different advantages and limitations of the methods. Therefore the combination of dissimilar approaches is demanded as an alternative way in order to overcome the limitations of standalone methods through complementary integration. Second, sparse linear regression that is penalized by the regularization parameter (lasso) and bootstrapping-based sparse linear regression methods were suggested in state of the art methods for network inference but they are not effective for a small sample size data and also a true regulator could be missed if the target gene is strongly affected by an indirect regulator with high correlation or another true regulator.

**Results:**

We present two novel network inference methods based on the integration of three different criteria, (i) z-score to measure the variation of gene expression from knockout data, (ii) mutual information for the dependency between two genes, and (iii) linear regression-based feature selection.

Based on these criterion, we propose a lasso-based random feature selection algorithm (LARF) to achieve better performance overcoming the limitations of bootstrapping as mentioned above.

**Conclusions:**

In this work, there are three main contributions. First, our z score-based method to measure gene expression variations from knockout data is more effective than similar criteria of related works. Second, we confirmed that the true regulator selection can be effectively improved by LARF. Lastly, we verified that an integrative approach can clearly outperform a single method when two different methods are effectively jointed. In the experiments, our methods were validated by outperforming the state of the art methods on DREAM challenge data, and then LARF was applied to inferences of gene regulatory network associated with psychiatric disorders.

## Background

Inferring gene regulatory networks (GRN) from biological data is currently the most interesting area of the systems biology research aiming to elucidate cellular and physiological mechanisms. GRN inference, which is often referred to as reverse engineering, is a process in which the network structure that best represents the regulation relationship over gene expression data is estimated. An inferred GRN consists of nodes and edges representing genes and gene-gene regulatory interactions (activation or suppression) respectively. Once the regulation maps are constructed by identifying the interactions of genes from high-throughput data such as gene microarray [1], we can gain insight into complex biological process from the regulatory networks in order to discover biomarkers for a target disease and apply further it to drug design [2, 3].

Basically the inference method should be determined depending on both what kind of data such as gene expression, gene-Transcription Factor (TF) [4], or protein-protein interaction (PPI) [5] are used to infer and which type of network model, such as directed or indirected graph [6], we assume. In addition, we have to consider the case of data integration. Namely, not only individual data but also multiple data types together (i.e. integration of gene expression and gene-TF data [7]) can be used for more reliable inference [8, 9]. As an assumption in this work, we limit our inference methods for directed network with a single data type: gene expression data. In order to decipher regulatory interactions with gene microarray data, which provides the gene expression level regulated by the other genes directly or indirectly, the number of effective network inference methods have been proposed by employing a variety of computational and structural models based on boolean networks [10], Bayesian networks [11], information theory [12], regression model [13], and so on. Depending on the different approaches, however, the results tend to be irregular due to inherently different advantages and limitations of each of the inference solutions [14]. The results of the Dialogue on Reverse Engineering Assessment and Methods (DREAM) project [15] describe well the pros and cons of the different methods as well as how effectively they can work together when the advantages of all methods are integrated (but it does not mean any combination always outperforms any other standalone method). More specifically, we note that they conclude two points through the experiments that (i) there is a limit to a single criterion for continuous improvement of network inference research without the integration and (ii) specifically the bootstrapping (re-sampling) based regression method [16] is required to avoid overfitting in regression-based methods [15].

**Fig. 1:**
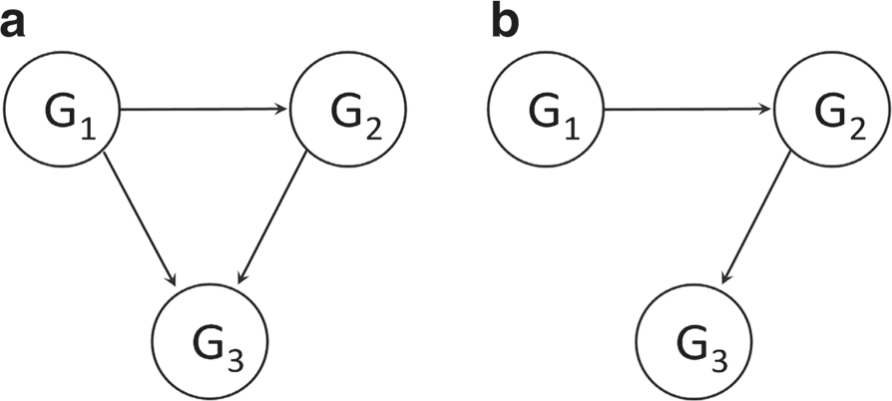
Example of network structures. **a** Feed-forward loops and **b** Cascades. When G3 is a target gene, G1 →G3 and G2 →G3 of Cascades are indirect and direct regulations respectively. In MI-based methods, indirect regulations are likely to be selected incorrectly in Cascades. In regression based method, strong direct regulators are more likely to be selected than another direct regulator in Feed-forward loops

As the motivation of our first strategy to this end, we focus on an integration of Mutual Information (MI) and L_1_ regularized linear regression referred to as lasso [17] but we exclude the learning Bayesian network in the integration. The learning structure of Bayesian networks is somewhat infeasible due to both the discretization problem of a small sample size data and the high cost of computational learning in large scale data. MI is an information theoretic criteria that has been conventionally used for learning large scale network structure [18]. Although MI based approaches such as CLR [19] and ARACNE [20] are limited to reconstructing only an indirected graph unlike linear regression and Bayesian networks, these methods have the popular advantages of computational simplicity and non-linear dependency enabler. In practice, the shortcoming of MI is that it is prone to fail in differentiation between indirect regulation and direct ones. For example, when there is a highly correlated indirect regulation from G1 to G3 like Fig. 1b, MI tends to incorrectly predict feed-forward loops (Fig. 1a) but not cascades (Fig. 1b). Lasso is also frequently used to select the regulators of a given target gene assuming sparseness of GRN in order to avoid the overfitting of the least-squares problem. In contrast to MI, indirect regulation edge in cascades could be pruned away by lasso in which the objective function is penalized for sparsity by a regularization parameter, called the tuning parameter *λ*. However, a weakness of regression-based method is that only a strong direct regulator is more likely to be selected than another direct regulator in Feed-forward loops. Therefore, the integration of two methods is considered to deal with the trade-off. The motivation of our second strategy is that the property of knockout data allows us to measure statistical variations between wild-type gene expression and perturbed gene expression after knocking them out to provide the cause-effect information between those two genes. However, there is the limitation that the method is only applicable to gene knockout data.

In this paper, we propose two methods, IMLARF (integration of MI and LARF) and ISLARF (integration of z-score and LARF). First, IMLARF indicates the integration of MI and LARF and consists of three steps. The first step of IMLARF is to build a matrix where each element is an edge score calculated by MI. In order to overcome the limitation of MI as mentioned above, the second step is to construct another edge score matrix using LARF, then the two edge score matrices are combined as the last step. In LARF, we regard a sparse linear regression as a feature selection since our goal is to identify the regulators that best predict the expression level of target genes. The problem is that features selected by lasso tend to be overfitted to a given tuning parameter *λ*, and thus the *unstability* problem caused by this overfitting can be solved by using bootstrapping [12, 21] in which data is randomly re-sampled so that a more stable selection can be achieved. However, the limitation of re-sampling is that it could not be effective in the case of a small sample size. Another limitation of bootstrapping is that the true variable (regulator gene) is likely to be missed (false negative) when strong indirect or direct regulators exist. LARF is similar to bootstrapping but LARF selects variables among randomly pre-selected candidate features in each iteration over different tuning parameters of lasso optimization so that true features weakly correlated to the target gene could not be missed, excluding indirect or direct regulators from the feature set. The second method we propose is ISLARF, which integrates two criteria, ZS and LARF. ZS is the name of the criteria that uses the z-score of variation of the knocked out gene expression. Although ISLARF is available only to knockout data, the performance is highly superior to other z-score based similar methods with knockout data in related works.

In the experimental evaluation, we validate the proposed method on a dataset from the DREAM3 challenge [22]. In addition, we explore the gene networks of Psychiatric disease with the related genes. The results shows that the proposed method significantly outperforms the state-of-the art [23, 24] and re-builds the known regulations of genes possibly associated with Psychiatric Disorders.

## Methods

### Problem definition

We begin with a brief definition of problems and notations. The network we target is a directed graph that consists of *n* nodes and *n*(*n*−1) edges representing genes and regulations respectively. Given a matrix $\mathbf {X}{\in }\mathbb {R}^{N{\times }n}$ where *N* is number of samples, we denote the *i*-th column by a vector **x**_*i*_ indicating expression levels of *i*-th gene over *N* samples, and we also let *X*={*X*_1_,…,*X*_*n*_} be a set of variables (genes, features, node, and variable are interchangeably used in this paper). The goal of our work is to not only identify the regulators given a target gene but also to define the confidence level of regulation as a weight of the edge. In other words, we estimate the weight of all possible regulations, which are directed edges between all pairs of nodes {*X*_*i*_←*X*_*j*_:*i,j*∈*X*} in the network, then select only edges that have a higher weight than pre-defined threshold *θ*. As a final result, therefore, a weight matrix $\mathbf {W}{\in }\mathbb {R}^{n{\times }n}$ is returned by the inference method, and ${W^{i}_{j}}$ represents a confidence level of the regulation when target gene *i* is connected to activator or suppressor gene *j*. In the following sections, we present how the edge weight is estimated by information theory, the LARF algorithm, and the z-score from knockout data.

### Overview

#### IMLARF and ISLARF

**Fig. 2:**
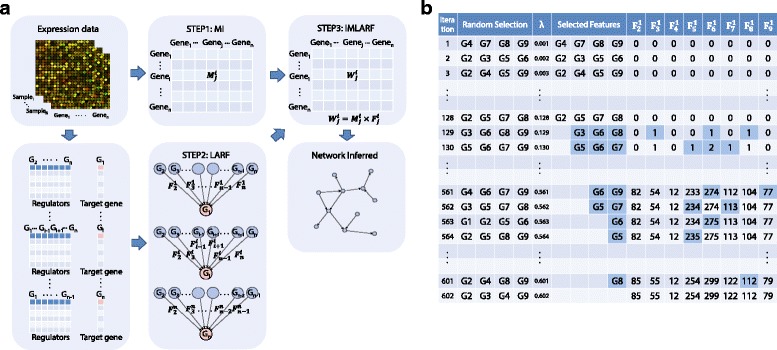
Overview of IMLARF and an example of LARF. **a** Overview of IMLARF. The algorithm consists of three steps, the construction of matrix (i) *M* and (ii) *F* and (iii) pairwise product of *M* and *F*. In ISLARF, the matrix *M* in step 1 is simply replaced with the matrix *S* (Section “Statistical approach”). **b** An example of procedures of LARF. It shows how the row vector *F* ^1^ of frequency matrix *F* given target gene G1 and 8 other candidate regulators (G2 ∼G9). By a predefined *α*, four random features are selected among eight genes in each iteration. In the beginning, *F* ^1^ is not increased and four random features are selected without sparsity since *λ* is not increased enough yet. The more *λ* is increased, the more the number of selected features (blue-colored cells) is decreased. If no feature is selected due to a highly increased *λ*, the iteration and frequency measure is finished

The first method we propose, IMLARF, consists of three steps. Figure 2a describes the overview of the proposed method. First, a symmetric edge weight matrix *M* is calculated by mutual information assuming that, if two genes have a higher mutual dependency, they are more likely to be in the regulation relationship. Second, another edge weight matrix *F* is produced by the LARF algorithm that consistently gives higher weight to the true edge from regulator to target gene. Lastly, the two weight matrices are combined by their entry-wise product $M{\circ }F=\{{M^{i}_{j}} \cdot {F^{i}_{j}}|i,j=1,\ldots,n\}$. The second method, ISLARF, is similar to IMLARF but using z-score matrix, *S*, is used instead of MI matrix. If ${S^{i}_{j}}$ has higher value, gene *i* is more likely to be regulated by gene *j*. So in the last step *S* is combined with *F* by their entry-wise product *S*∘*F*

### Information theoretic approach

#### Mutual information matrix

The dependency of two genes, *X*_*i*_ and *X*_*j*_, can be measured by MI defined as

1 $$ I(X_{i},X_{j})=\sum_{X_{i},X_{j}}p(X_{i},X_{j})\log\frac{p(X_{i},X_{j})}{p(X_{i})p(X_{j})},  $$

The strength of MI is the ability to measure non-linear dependencies of genes, but the limitation in practice is that the discretization of gene expression is required to calculate the probability of *X*_*i*_ and *X*_*j*_. Instead, if we assume the Gaussian distribution of gene expression, MI can be computed with its original continuous values by using Gaussian mutual information [25] defined as

2 $$ I(X_{i},X_{j})=-\frac{1}{2}\log\frac{|cov(X_{i},X_{j})|}{|cov(X_{i},X_{i})||cov(X_{j},X_{j})|},  $$

where *cov*(*X*_*i*_) is the covariance matrix of variable *X*_*i*_, and |*cov*| is the determinant of covariance matrix. The reader is referred to [26] for more details. We build MI matrix in which each element ${M^{i}_{j}}$ indicates the dependency between *X*_*i*_ and *X*_*j*_ which means that *X*_*i*_ and *X*_*j*_ are independent if ${M^{i}_{j}}=0$ or ${M^{i}_{j}}$ is relatively lower than other edges. Networks with the edges whose ${M^{i}_{j}}$ are higher than the heuristic threshold are referred to as relevance networks. Two critical limitations of relevance networks, however, are that firstly, MI does not provide the direction of edges due to ${M^{i}_{j}}={M^{j}_{i}}$, and secondly, the high co-expression and indirect regulation may cause false positives.

### Statistical approach

#### Z-score and gene knockout data

We note that knockout data implies cause-effect information. The gene expression level after the perturbation of another certain gene provides the chance to observe if the gene is downstream of the perturbed gene. For example, if the variation between wild type of gene *j* ($X^{wt}_{j}$) and gene *j* expression measured after gene *i* is knocked out is high, gene *j* is likely to be regulated by gene *i*. The variation matrix *D* is defined as

3 $$ {D^{i}_{j}}=X^{-i}_{j}-X^{wt}_{j}  $$

4 $$ {S^{j}_{i}}=\left|\frac{{D^{i}_{j}}-\mu_{D_{j}}}{\sigma_{D_{j}}}\right|  $$

where $X^{-i}_{j}$ is the expression level of gene *j* after knocking gene *i* out, and $\mu _{D_{j}}$ and $\sigma _{D_{j}}$ is mean and standard deviation of *j*-th column vector *D*_*j*_ of variation matrix *D* respectively. As the z-score of ${D^{i}_{j}}$ over *D*_*j*_ is the weight of regulation edge *Gi*→*Gj*, the z-score of ${D^{i}_{j}}$ is equivalent to ${S^{j}_{i}}$ of edge weight matrix S. The limitation of this criterion is the availability only in knockout data.



### LARF algorithm

The third approach for complementary integration of inference methods is based on L_1_-regularized linear regression (lasso) defined as

5 $$ argmin_{\beta} ||X_{i}-X^{\setminus{i}}\cdot\beta_{i}||_{2}^{2}+\lambda||\beta_{i}||_{1}  $$

where coefficient column vector *β*_*i*_ represents regulation relationships between the target gene *i* and others. More precisely, after *β*_*i*_ is optimized to minimize the objective function (5), then if the *j*-th element of *β*_*i*_ is zero, gene *j* does not regulate gene *i*, otherwise it does. The optimization is performed for each target gene *i*, *i*∈*X*. Coefficient matrix *B*={*β*_1_,…,*β*_*n*_}^*T*^ is equivalent to adjacency matrix where non-zero *B*_*ij*_ is the regulation edge from regulator gene *j* to target gene *i*. The tuning parameter *λ* in lasso is used to enforce network sparsity, so the number of selected (non-zero coefficient) variables varies with different *λ*. In our works, we regard variable selection of lasso as a feature selection to predict a target gene’s expression level.

To overcome the overfitting problem and the strong indirect regulation problem, lasso is iteratively performed over different *λ* with randomly pre-defined candidate features rather than random samples like bootstrapping. More precisely, the basic idea of LARF is that lasso is iteratively performed with only randomly selected candidate features while increasing the tuning parameter, then giving weight to each feature by counting how many times each feature is selected in the iterations. We predefine the fraction of the number of all possible features as a parameter *α* (0<*α*<1) for the candidate features. For example, when the number of all possible regulators is *n*=100, *α*=0.2 means that only 20 random candidate genes are used in a single iteration of lasso. After random featuring, random sampling is performed with parameter *r* which decides how many samples are used from the original data. For instance, when the original sample size is *N*=200 and *r*=0.7, only 140 random samples are used in each iteration of lasso. With randomly (uniform distribution) selected features and samples by parameter *α*, we iteratively run lasso over increasing tuning parameter *λ* until lasso does not select any features due to a certain high *λ*. In each iteration, random candidate features and samples are re-defined again. Tuning parameter starts from zero and increases by the parameter *stepsize* that should be small enough, (e.g 0.001). Otherwise, both re-featuring and re-sampling will be biased. For each iteration, the frequency matrix *F* is updated. The *i*-th row of *F* is the frequency of feature selection for target gene *i* (${F^{i}_{i}}$ is supposed to be zero). For example, Fig. 1b describes how the *F*^*i*^ is measured. After finishing the iterations (repeat in line 5), we iteratively perform *t* times (*t*=10 in our experiments) of the process from line 5 to 13 again, and then *i*-th row vector of the frequency matrix is normalized by

6 $$ {F^{i}_{j}}=\frac{({F^{i}_{j}}-min(F^{i}_{-i})}{max(F^{i})-min(F^{i}_{-i})},  $$

where

7 $$ F^{i}_{-i}=\{{F^{i}_{j}},j=1,\,i-1,i+1,\ldots n \},  $$

and *max*(*F*^*i*^) and $min(F^{i}_{-i})$ is maximum value of *i*-th row vector of *F* and minimum of $F^{i}_{-i}$.

## Results

We first evaluated the performance of IMLARF and ISLARF on synthetic simulation data as compared to the state of the art, and then explored the inferred networks with real gene microarray data for psychiatric disorders. The synthetic, non-linear expression data is from DREAM3 *In Silico* Network challenge in which the data is created with the subnetworks of well-known reference networks for *Yeast*. To assess the edge weight matrix *W* elicited by proposed methods, first the matrix is converted to an edge list sorted by the confidence levels (weight), then the top *k* confidence level edges are selected to measure the accuracy criteria, such as true positive (TP), false positive (FP), true negative (TN), and false negative (FN). The receiver operating characteristic (ROC) curves as a parametric curve were traced over different *k*=1,...,*n*(*n*−1) to examine the trade-off between the true positive rate (TPR) and false positive rate (FPR). The criteria to represent the performance are defined as following:

- TPR=TP/(TP+FN)
- FPR=FP/(FP+TN)
- AUROC: the area under ROC curve.

We compared our method to each standalone method without integrations and also other well known the state of the art methods. The abbreviations of algorithms are listed below:

- MI: edge is scored by mutual information
- ZS: relative variation from wild type is measured by z-score.
- LARF: lasso based random featuring and sampling.
- IMLARF: integration of MI and LARF
- ISLARF: integration of ZS and LARF
- ZDR: top rank in DREAM 3 [23]
- GENIE3: top rank in DREAM 4 [24]
- TIGRESS: top rank in DREAM 5 [21]

### Evaluation on the DREAM3 benchmarks

#### Materials

The data for DREAM3 *In Silico* Network challenge consists of three differently sized networks, (10, 50, and 100 genes), and there are five gold-standard networks for each size (total of 15 networks). The five networks are named Ecoli1, Ecoli2, Yeast1, Yeast2, and Yeast3. From each true network, three different data types (knockdown, knockout perturbations, and time series data) are provided, and the knockdown and knockout data includes a single wild type sample. In our experiments, only knockout data is used and 10-gene, 50-gene, 100-gene of Yeast1 networks are mainly tested.

#### Random sampling vs Random featuring

**Fig. 3:**
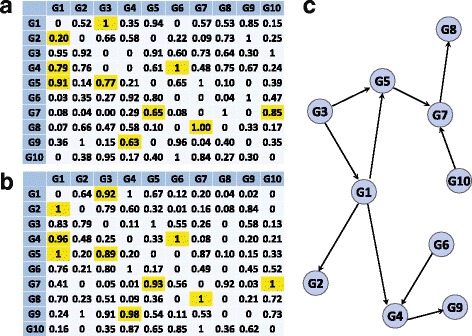
Comparison of random sampling and featuring in LARF. **a** The result of LARF with only random sampling. **b** The result of LARF with only random featuring. **c** True network of 10-gene Yeast1 in DREAM3

To evaluate how much more effectively LARF selects true edges than random sampling, we compared them with 10-gene Yeast1 network in Fig. 3. Figure 3a is the result of LARF with only random sampling (*α*=1, *r*=0.5) and 3b is with only random featuring (*α*=0.5, *r*=1). The normalized edge score is the average of 10 experiments and yellow colored cells indicate true edges. In Fig. 3a, though G2’s true regulator is G1, G2 ←G3 is relatively higher than G2 ←G1 probably because of indirect regulation from G3 to G2 through G1. In Fig. 3b, G2 ←G1 is correctly estimated as true edge by random featuring. Similarly two true edges (G4 ←G1 and G5 ←G1) are inferred with the highest weight in random featuring but random sampling gives only 0.79 and 0.91 to two true edges (G4 ←G1 and G5 ←G1) due to another true edges (G4 ←G6 and G5 ←G3) have strong direct regulation (1 and 0.99).

#### Setting parameters

**Fig. 4:**
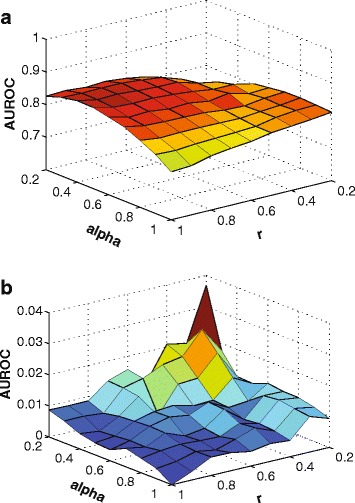
Mean and standard deviation of AUROC with different parameters and 10 iterations of experiments for 50-gene Yeast1 network. (**a**) Mean and (**b**) standard deviation

Before we compare our methods to other methods, we explored the optimal parameters that give the best results. As described in Fig. 4, the mean and standard deviation of AUROC are measured after LARF are 10 times performed over different parameters, *α* and *r*, for 50-gene Yeast1 network. The range of parameter is 0.2 ∼1 due to too small number of feature and sample in 10-gene network data. The best result (0.8501 ±0.0049) is recorded with *α*=0.4 and *r*=1 for 50-gene Yeast1 data. This indicates that the random sampling rate does not necessarily need to be applied to avoid overfitting once random featuring is applied. In addition, the figure also shows that the AUROC can be decreased with high standard deviation if both parameters are too small. According to the result of 10-gene and 100-gene Yeast1 data, if the sample size is small (*N*=10), the deviation is quite high in low *α* and *r* though AUROC is high. As the best result for 10-gene and 100-gene Yeast1 data, 0.925 ±0.0125 and 0.8611 ±0.0046 were achieved with *α*=0.5, *r*=1 and *α*=0.4, *r*=1 respectively. It also shows the random sampling could not make an improvement in both small and large sample sizes. Therefore we applied fixed parameters *α*=0.5, *r*=1 to all data sets in our experiments.

#### Effect of integration and performance comparisons

**Fig. 5:**
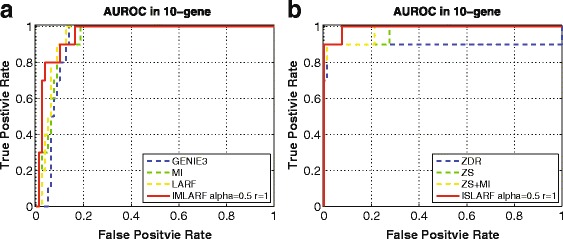
ROC of the methods (**a**) without and (**b**) with gene deletion information in 10-gene network

Table 1 presents the performance of integrative approaches compared to a single method. In the case of LARF-based methods, mean and deviation are measured after each method is performed 10 times for Yeast1 network of DREAM3. The integration of more than two methods is simply done by entry-wise product of edge score matrix. In TIGRESS-TF, the list of TF is provided as TIGRESS is designed for DREAM5 challenge data in which TF is given. Asterisk(*)-marked methods require knockout data. The integration of MI and LARF outperforms standalone MI and LARF except 50-gene. Similarly the performance of ISLARF is better than other integration such as ZS+MI and standalone ZS. If knockout data is not available, IMLARF will be the best method as ZS is not applicable. Since ZDR is based on knockout data, the result shows that ZDR is quite better than other methods such as IMLARF except in a small size network. In Fig. 5, the AUROC for proposed methods and the state of the art methods with 10-gene Yeast1 data are plotted after only a single experiment. Overall results show that ISLARF is the best method if knockout data is available, otherwise IMLARF is superior to other methods.

**Table 1:** AUROC of standalone and integrative methods

Method	10-gene	50-gene	100-gene
GENIE3	0.9175	0.8427	0.8631
TIGRESS	0.7044 ± 0.0056	0.8179 ± 0.0025	0.7690 ± 0.0023
TIGRESS-TF	0.8154 ± 0.0037	0.9006 ± 0.0010	0.8777 ± 0.0009
MI	0.9312	0.8329	0.8586
LARF	0.9250 ± 0.0154	**0.8489** ± 0.0038	0.8610 ± 0.0039
**IMLARF**	**0.9425** ± 0.0047	0.8487 ± 0.0032	**0.8701** ± 0.0012
ZDR ^∗^	0.8975	0.9223	0.8876
ZS ^∗^	0.9725	0.9204	0.8870
ZS ^∗^+MI	0.9775	0.8931	0.8925
**ISLARF** ^**∗**^	**0.9892** ± 0.0021	**0.9301** ± 0.0049	**0.9065** ± 0.0029

### Inference of GRN for psychiatric disorders

In this section, the proposed method is applied to real gene expression data for psychiatric disorders. Through the experiments, we evaluate how the method constructs the network and explore what potential biomarkers of Psychiatric disorders are in the inferred networks. Psychiatric disorders data that are provided from the Stanley Medical Research Institute (SMRI) consist of gene expression data of 25833 genes and 131 samples (43 controls and 88 cases) including bipolar disorder, schizophrenia, major depression as three major psychiatric diseases.

**Fig. 6:**
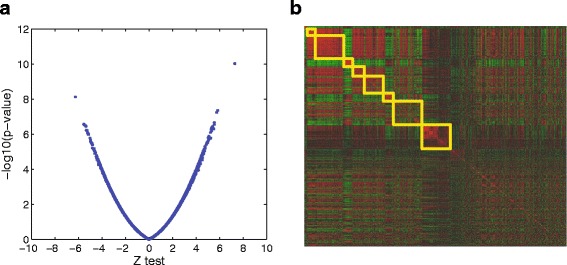
Statistical test and clustering for gene selection. **a** t-test and z-test **b** clustered correlation matrix and 8 clusters (*yellow squares*)

To select genes possibly associated with psychiatric disorders, two statistical tests, *t*-test and *z*-test [27], are performed. In Fig. 6a, all genes are plotted by using *p*-value of *t*-test for y-axis and *z*-test value for x-axis, and the plot shows that two tests shows similar results in linear patterns. From these two tests, we selected 1407 genes as cut-off values are set to −*log*_10_(0.01) and ±2.326 for *t*-test (y-axis) and *z*-test (x-axis). To find a module of genes that may interact to each other in Psychiatric disorders, we initially built a correlation matrix whose element of *i*th row and *j*th column is absolute value of correlation between expressions of *i*th and *j*th genes, and then clustering is performed to the estimated correlation matrix as shown in Fig. 6b. Based on the result of clustering, we manually set 8 groups of genes (yellow squares).

**Fig. 7:**
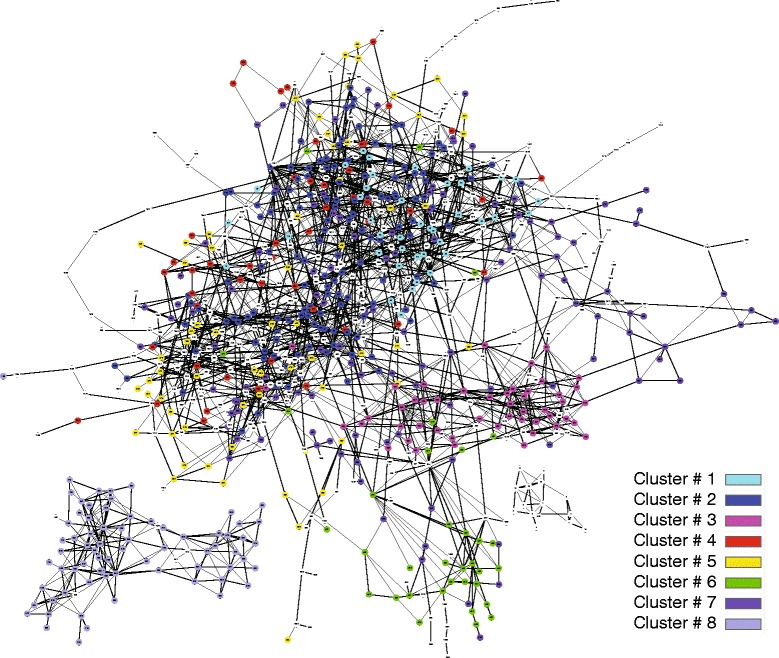
Large components of network inferred with 1407 genes

To analyze the relationship between clusters, first, IMLARF was applied to all 1407 genes with setting *θ* to 0.2. Figure 7 shows only the two largest components of the inferred network where node color indicates a cluster number after small components of the network are removed from the figure. The result is consistent with the correlation matrix in Fig. 6b showing the features as follows: (i) cluster 3, 6, and 8 in the network strongly and exclusively interact to each other, (ii) cluster 2, 4, and 5 are complicatedly interacting together, (iii) cluster 7 is widespread over the whole network.

**Fig. 8:**
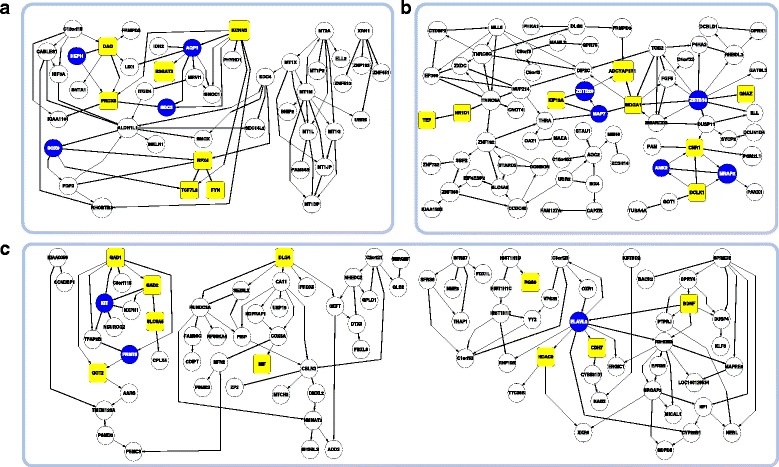
Inferred gene regulatory networks for psychiatric disorder. **a** Cluster 3 **b** Cluster 7 **c** Cluster 4 and 5. Yellow-colored nodes indicate the genes known as psychiatric disorder genes in the literatures. Blue-colored nodes are the genes that are connected to more than two yellow genes

To observe the strong regulation of the network, we inferred network with all the genes again after setting *θ* to 0.4. As a result, we displayed the second largest component in the inferred network in Fig. 8a. Most nodes of the network are genes of cluster 3 implying that cluster 3 is most exclusively and strongly interacting within the cluster. It is noted that 7 genes, DAO [28], PRDX6 [29], KCNN3 [30], TCF7L2 [31], RFX4 [32], FYN [33], and B3GAT2 [34] (yellow-colored nodes), relevant to psychiatric disorders are involved and interestingly these genes except B3GAT2 constitute a connected subgraph. Blue-colored nodes indicate the genes that have more than two connection to yellow nodes supposing that these genes are likely to be susceptible to psychiatric disorders (In this paper we call yellow and blue gene reference gene and susceptible gene respectively. We define a gene as a reference gene if a gene appears with a psychiatric disease in the title of related literatures). There are 4 genes, SOX9, HEPH, AQP1, and SDC3 as susceptible genes, and it was already reported that SDC3 has a weak association with schizophrenia in related GWAS [35].

Figure 8b is the inferred network for cluster 7, and a total of 8 genes known as psychiatric disorder-related genes in related literatures are found as following: TEF [36], NR1D1 [37], KIF13A [38], ADCYAP1R1 [39], MDGA1 [40], GNAZ [41], CNR1 [42], and DCLK1 [43]. Additionally we defined 5 genes, ZBTB20, MAP7, ZBTB16, ANK2, and MRAP2, as susceptible genes, and surprisingly ZBTB20 [44], MAP7 [45], ZBTB16 [46], ANK2 [47] was also reported as schizophrenia disorder-associated genes in SNP and CNV-based studies. So we imply that it is worth to investigate the genes that have only an edge to reference gene as candidate genes associated with psychiatric disorder. In addition, reference genes in the network tend to interact with each other directly or indirectly though susceptible genes but they are not widely spread implying they may work together or may be co-regulated by another unknown biomarker.

The network inference result for the combination of cluster 4 and 5 is shown in Fig. 8c consisting of two components. There are 10 reference genes such as DLG4 [48]], MIF [49], SLC6A5 [50], GAD1 [51], GAD2 [52], GOT2 [53], RGS9 [54], HDAC9 [55], CDH7 [56], and BDNF [57], and 3 susceptible genes such as PRMT8, KIT, and ELAVL2. It is noted that ELAVL2 has connections to three reference nodes and was reported as schizophrenia-related gene in recent GWAS [58].

## Discussion

The difference between ZS and z-score of [23] is in whether the absolute value of variation ${D^{i}_{j}}$ is taken before z-scoring or original value of ${D^{i}_{j}}$ is used. In our method, we simply calculate the z-score to measure how many deviations the observed variation is above or below while the absolute value of variation $|{D^{i}_{j}}|$ is used for z-score. Since we want to know how much the variation of a gene is higher than another target gene after knockout of the source gene, the use of ${D^{i}_{j}}$ rather than $|{D^{i}_{j}}|$ is more reasonable and it is not guaranteed to select high-variant genes if absolute value of ${D^{i}_{j}}$ is used. Since random featuring and random sampling are performed in iterations of lasso, the computational time is significantly increased especially in finding optimal parameters. In implementation, the step size, therefore, should be set to a reasonably small value, and parallel processing (i.e. *parfor* in matlab) can reduce the processing time in practice (In our case, eight local cores are used). As a future work, we can integrate TF information additionally in the inference so that we can get more reliable results, and then also apply our method to DREAM5 challenge data for comparison to TIGRESS that utilizes TF information.

## Conclusion

We presented two integrative approaches for gene regulatory network inference combining two different algorithms. First, IMLARF that we proposed is based on the integration of MI and LARF, which is a novel regression-based random featuring, to overcome the limitation of random sampling and MI. Secondly, ISLARF is the combination of LARF and ZS that is based on the z-score of variation of expression after the candidate regulator is knocked out. Both integrative methods outperform the standalone methods and the selected state of the art techniques on DREAM3 challenge data. In application to inference of the gene regulation associated with psychiatric disorders, we applied IMLARF to gene expression data and inferred the interactions between genes reported known as psychiatric disorder-associated genes and susceptible genes defined by inferred networks.
